# The effect of self‐designed metabolic equivalent exercises on cancer‐related fatigue in patients with gastric cancer: A randomized controlled trial

**DOI:** 10.1002/cam4.7085

**Published:** 2024-05-08

**Authors:** Xiao Xin, Lei Huang, Qi Pan, Jun Zhang, Weiguo Hu

**Affiliations:** ^1^ Medical Center on Aging of Ruijin Hospital, MCARJH Shanghai Jiaotong University School of Medicine Shanghai China; ^2^ Department of Oncology, Ruijin Hospital Shanghai Jiao Tong University School of Medicine Shanghai China; ^3^ Department of Nursing, Ruijin Hospital Shanghai Jiao Tong University School of Medicine Shanghai China; ^4^ Department of Geriatrics, Ruijin Hospital Shanghai Jiao Tong University School of Medicine Shanghai China; ^5^ Department of Surgery, Ruijin Hospital Shanghai Jiao Tong University School of Medicine Shanghai China

**Keywords:** cancer‐related fatigue, exercise intervention, quality of life, self‐designed metabolic equivalent exercises

## Abstract

**Aims:**

To investigate the effect of Self‐designed Metabolic Equivalent Exercises (SMEE) on cancer‐related fatigue in patients with gastric cancer.

**Methods:**

130 patients with gastric cancer admitted to Department of Oncology of a tertiary hospital in Shanghai were enrolled and assessed for eligibility. After excluding 1 patient who declined to participate, 129 eligible patients were randomly assigned into SMEE (*n* = 65) and control (*n* = 64) groups. The Revised Piper Fatigue Scale (RPFS) and EORTC QLQ‐C30 Quality of Life Scale were used to measure cancer‐caused fatigue and quality of life, respectively, in both groups at the first admission and after 3 months.

**Results:**

After excluding patients who did not receive allocated intervention due to medical (*n* = 3) and personal (*n* = 2) reasons, those who were lost to follow‐up (*n* = 3), and those who had discontinued intervention (*n* = 2), 119 patients (64 in the SMEE group and 55 in the control group) were included for analysis. There were no statistically significant differences in the RPFS or QLQ‐C30 score between the two groups at baseline. After 3 months, the total RPFS score of the SMEE group was significantly lower than that of the control group (2.86 ± 1.75 vs. 4.65 ± 1.29, *p* = 0.009), with significant improvements in affective meaning (0.83 ± 0.92 vs. 1.13 ± 0.77, *p* = 0.044) and sensory (0.70 ± 0.71 vs. 1.00 ± 0.54, *p* < 0.001) subscales; in the SMEE group, QLQ‐C30 scores in somatic (2.00 ± 0.27 vs. 1.31 ± 0.26, *p* < 0.001), emotional (2.67 ± 0.58 vs. 2.07 ± 0.48, *p* < 0.001), and social (3.23 ± 0.58 vs. 1.64 ± 0.51, *p* < 0.001) functioning were significantly higher than those in the control group, with significant improvements in fatigue (*p* < 0.001), nausea/vomiting (*p* = 0.014), shortness of breath (*p* < 0.001), constipation (*p* < 0.001), and diarrhea (*p* = 0.001) dimensions.

**Conclusion:**

The self‐programmed metabolic equivalent manipulation as an exercise intervention could effectively reduce the degree of cancer‐caused fatigue and improve quality of life in patients with gastric cancer.

## Graphical abstract

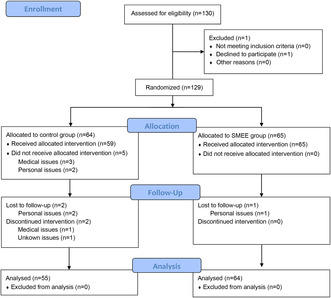

## 1 INTRODUCTION

The latest report from GLOBOCAN indicated that there were approximately 1.08 million new cases of gastric cancer (GC) in the world in 2020.^1^, ^2^ In China, approximately 470,000 new cases are diagnosed with GC yearly, accounting for about 8% of all cancer incidences.^3^ With advancements in medical technology, the cure and survival rates for patients with GC are increasing year by year.^4^, ^5^, ^6^, ^7^ Therefore, more attention should be paid to the quality of life of patients with GC.^8^ Cancer‐related fatigue (CRF) is one of the important factors affecting the quality of life of patients with GC.^9^

Fatigue is a symptom expressed by cancer patients through all stages of the disease trajectory. CRF is a painful, persistent, subjective feeling of physical, emotional, or cognitive exhaustion or weakness that is inconsistent with the amount of recent activity. It is associated with cancer disease or treatment and interferes with daily function.^10^ 75%–96% of patients undergoing chemotherapy and 75%–100% of patients receiving radiotherapy have different degrees of CRF.^11^ The overall prevalence of CRF is roughly 49% across various cancer populations, depending on tumor type, cancer stage (61% in advanced cancers), and treatment status (62% during treatment).^12^ CRF seriously affects the physiological function and psychological state of patients with GC and becomes an important factor negatively affecting the treatment, rehabilitation, and quality of life of patients.

Despite its high prevalence and debilitating effects, the etiology underlying CRF remains not well understood. CRF may be linked to complex multifactorial processes, including a range of molecular/physiological, inflammatory/immunological, hypothalamic–pituitary–adrenal (HPA) axis‐associated, and psychological factors, as well as reduced energy metabolism, comorbidities, and cancer treatment.^12^, ^13^, ^14^ Studies have shown that exercise is an effective nonpharmacological intervention to treat CRF.^11^, ^13^, ^15^ Exercise intervention can effectively alleviate CRF symptoms in patients with breast cancer, colorectal cancer, lung cancer, prostate cancer, or other cancers.^16^, ^17^ As confirmed by the Evidence‐based Medicine Group of the American Cancer Society, the National Comprehensive Cancer Network (NCCN), the Oncology Nursing Society, the Canadian Cancer Society, and the American Society of Clinical Oncology, exercise is an effective intervention measure against CRF.^15^, ^18^ Exercise intervention has been explicitly recommended as the first‐level evidence for the management of CRF in the Chinese Clinical Practice Guidelines for Cancer‐Related Fatigue (2021 Edition).^19^ There are few reports on the effective use of exercise intervention in GC patients.

Evidence on the most effective approach of physical training interventions to combat CRF remains fragmented. Zhang et al.^20^ showed that Tai Chi was an effective intervention for managing CRF in patients with lung cancer undergoing chemotherapy, especially for decreasing general fatigue and physical fatigue, and increasing vigor. Another study indicated that Baduanjin exercise was an effective and appropriate intervention for improving quality of life and was worthy of recommendation and implementation during the rehabilitation process in patients with nasopharyngeal carcinoma.^21^ There have been few randomized interventional studies on CRF in patients with GC in China or other countries.

Before this randomized controlled trial (RCT), we found through a cross‐sectional survey study that only 35% of GC patients continued adhering to exercise after developing cancer, that 79% of patients would like to receive professional guidance on exercise during their homestay period, including the way, intensity, and time of exercise, and that notably, 93% of patients had concerns about the safety of exercise (unpublished data). Based on this, it is necessary to develop a suitable exercise intervention for GC patients at home with ensured safety and quantifiable intensity. The design of the metabolic equivalent exercises was based on the exercise oncology prescription framework proposed by Sasso et al.^17^ and incorporated three dimensions: Pre‐exercise assessment, exercise program development principles, and exercise prescription design. The choreography of the exercises combined with radio gymnastics, which is very familiar to the Chinese population, and was in line with the principles of individualization, targeting, rest and recovery, and progressive overload. Based on an evidence‐based approach, this study investigated the effect of the Self‐designed Metabolic Equivalent Exercises (SMEE) program on CRF in patients with GC in a randomized controlled setting.

## 2 METHODS

### 2.1 Patients

Patients with GC admitted to the Department of Oncology, Ruijin Hospital, Shanghai Jiao Tong University School of Medicine, a Class A tertiary hospital in Shanghai, China, between April 2020 and April 2021 were enrolled. The inclusion criteria were as follows: Patients with GC (1) Aged 18 years or older; (2) with clear pathological diagnosis and staging; (3) undergoing chemotherapy; (4) with CRF based on a total score ≥1 on the Revised Piper Fatigue Scale (RPFS) on admission; (5) with a Barthel index greater than 80 points; (6) who agreed to participate voluntarily and who signed an informed consent form; and (7) with expected survival time of greater than 3 months. The exclusion criteria were as follows: Patients (1) with heart, lung, liver, kidney, or other vital organ failure; (2) with mental disorders and unable to communicate verbally; and (3) with sudden changes in the disease course. This randomized controlled trial (RCT) was registered in clinicaltrials.gov (NCT05401045), and followed the CONSORT guidelines.

### 2.2 Research tools

#### 2.2.1 General information questionnaire

The general information questionnaire collected the following data: Age, sex, education, residence status, marital status, height, body weight, body mass index (BMI), Barthel index, disease diagnosis, stage, previous exercise frequency, exercise time, and type of exercise.

#### 2.2.2 Exercise records

Every patient recorded the following information pertaining to exercise: Start and end time, date, type, and intensity of exercise. The recorded contents included the date, duration, heart rate after exercise, and self‐measured pulse for 1 min. Each patient was provided with a follow‐up manual for cancer patients that contained education materials regarding CRF, a QR code for a metabolic equivalent exercise video, and exercise plan implementation form.

#### 2.2.3 Primary endpoint

The primary endpoint of this study was the total score of the Revised Piper Fatigue Scale (RPFS) for measuring the cancer‐related fatigue (CRF) status. The RPFS includes 22 items and 3 open‐ended questions regarding the duration of fatigue, the possible causes of fatigue, fatigue‐influencing factors, measures to relieve fatigue, and symptoms related to fatigue.^7^ The 22 items address the degree that fatigue affects daily activities (6 items), emotional (5 items) and physical (5 items) factors that affect fatigue, and the cognitive and emotional statuses of the respondent (6 items). A number from 0 to 10 is used to indicate the degree of fatigue, with 0 indicating no fatigue and 10 the most severe fatigue; the higher the score is, the more severe the fatigue is. In 2003, So et al.^22^ translated the RPFS into Chinese; the test–retest reliability was 0.98, the Cronbach coefficient α of the 4 subscales ranged from 0.89 to 0.93, and the Cronbach coefficient α of the total scale was 0.91.

#### 2.2.4 Secondary endpoint

The secondary endpoint was quality of life as measured by the European Organization for Research and Treatment of Cancer (EORTC) QLQ‐C30 scale. It is the core scale for measuring the quality of life of patients with cancer. It has been used to measure the quality of life of patients with various types of cancers. The Cronbach's coefficient α is 0.814. It has been widely used in many countries and/or regions and is currently the universal quality of life scale for patients with cancer. Wan et al. have used the Chinese version of the scale to assess patients with cancer in China. The test–retest reliability of the 15 domains were all above 0.73, and the α values of the internal consistency in each domain were all above 0.50, thus indicating that the scale could be used to determine the quality of life of patients with cancer in China. The scale has a total of 30 items that are evaluated in 5 functional scales (physical, role, emotional, cognitive, and social functioning). Except for Items 29 and 30, which address the overall health status, the other items are reversely scored, that is, the higher the score is, the worse the function and symptoms are. For scoring, the options for Items 1 to 28 are “no”, “a little bit”, “some”, and “a lot” with scores of 1, 2, 3, and 4, respectively; the scores for Items 29 and 30 range from 1 to 7. The raw score (RS) is first obtained and then converted to the standard score (SS). For the functional domain, SS = [1 – (RS – 1)/R] × 100; for the symptoms and general health status domains: SS = [(RS – 1)/R] × 100. The higher the functional field and overall scores are, the higher the quality of life of the respondent is; the higher the symptom field scores are, the worse the quality of life is.

### 2.3 Research methods

This study was a single‐center, open‐label, randomized controlled trial. The participants were randomly assigned into a SMEE or a control group in a 1:1 ratio according to a computer‐generated sequence.

#### 2.3.1 For the SMEE group (Video S1)

(1) The general information questionnaire, RPFS, and the Chinese version of the EORTC QLQ‐C30 (V3.0) were used for the baseline assessment of patients, and intervention was performed for patients with an RPFS score greater than or equal to 1.

(2) Exercise plan: Each session of the SMEE program was divided into 8 components: Stretching exercises, chest expansion exercises, kicking exercises, lateral movement exercises, body rotation exercises, whole‐body exercises, jumping exercises, and a cooldown. There were 4 sets and 8 repetitions per component, taking approximately 4 minutes to complete and consuming approximately 18 calories. Patients were instructed to exercise once in the morning and once in the evening. For patients with moderate fatigue and with an RPFS score of 4–6 points, low‐intensity exercises were recommended, that is, patients could choose 1–4 metabolic equivalent exercises and repeat them twice; for patients with mild fatigue and with an RPFS score of 1–3 points, moderate‐intensity exercises were recommended, that is, patients could choose to complete the entire set of metabolic equivalent exercises or 5–8 of the exercises and repeat them twice. The exercise frequency was 5 times per week. Nurses informed the participants of the precautions for exercises to ensure safe implementation.

Metabolic equivalent intensity: The intensity of exercises was expressed as metabolic equivalents (METs). For this study, greater than or equal to 6 METs indicated high intensity, 3–5.9 METs moderate intensity, and less than 3 METs low intensity.

(3) Exercise training: Members of the Fatigue Management Team in the ward taught the patients to perform metabolic equivalent exercises using videos. Exercise guidance was provided after assessments of surgical, catheter, and incisional pain. Patients could follow the department's WeChat public account to watch complete videos pertaining to metabolic equivalent exercises and related exercise precautions. The Fatigue Management Team members confirmed that a patient could perform the exercise independently and correctly.

(4) Health education: The participants were provided with information related to CRF (causes, clinical manifestations, associated factors, the necessity and importance of fatigue prevention, and measures to reduce CRF, etc.) and exercises (intensity, time, frequency, precautions, etc.)

(5) Recording: Each participant completed a form after each exercise session.

(6) Follow‐up: A nurse followed up with each patient by telephone every 2 weeks to determine if the patient completed his or her exercise sessions. Exercise completion rate (%) = (actual exercise time ÷ planned exercise time) × 100%. Nurses supervised and provided reminders to patients with completion rates below 50%. In addition, each patient's exercise success rate was calculated: Exercise success rate (%) = (real‐time heart rate after exercise/target heart rate) × 100% (>70% was considered to be up to standard). Target heart rate = (200‐age) × 100%; a heart rate of 70%–80% of the target heart rate could improve cardiopulmonary function.

#### 2.3.2 For the control group

Participants received routine exercise health education that included information pertaining to CRF (causes, clinical manifestations, associated factors, the necessity and importance of fatigue prevention, and measures to reduce CRF, etc.) and exercises (3–5 times per week, regardless of the type of exercises). The patients were also informed of the precautions for exercise.

After 3 months, the SMEE and the control groups were reassessed using the RPFS and the Chinese version of the EORTC QLQ‐C30 (V3.0).

### 2.4 Data collection

The enrolled patients had a face‐to‐face interview to complete the questionnaires. The two data collection timepoints were before intervention (T0) and 3 months after intervention (T1). The patients in the SMEE group received a follow‐up call every 2 weeks to check his or her exercise performance.

### 2.5 Statistical analyses

The sample size was calculated using the GPower sample size calculation software; α was set to 0.05, and 1–β to 0.80, resulting in a calculated sample size of 54 patients in either group to achieve a 2‐point decrease in the primary endpoint, the total score of the Revised Piper Fatigue Scale (RPFS). Considering a sample loss rate of 15%–20%, at least 65 patients were needed in each group. A total of 130 patients were enrolled in this study.

The SPSS 26.0 software was used for the statistical analyses. Measurement data were expressed as mean ± standard deviation (SD), and data with a non‐normal distribution as median (interquartile range). The *t* test was used for comparison of data with a normal distribution between two groups, and the Wilcoxon rank‐sum test was used for comparison of data with a non‐normal distribution. Categorical data were expressed as count (frequency [%]), and the chi‐square test was used to determine differences between groups. To address missing data, we performed both intention‐to‐treat (ITT) and per‐protocol (PP) analyses. Since the findings were based on continuous data (i.e., fatigue and quality of life scale scores), only complete‐case analyses were applicable to them. The randomized controlled design of this study would make confounding variables comparable between the SMEE and control groups. *p* < 0.05 was considered statistically significant.

### 2.6 Ethics approval

This study was conducted in accordance with the Declaration of Helsinki and approved by the local Ethics Committee of Ruijin Hospital, Shanghai Jiao Tong University School of Medicine. The attributes, benefits, uses, and disadvantageous effects of the study were all explained to all participants and informed consent was obtained from all individual participants included in this study. Patients had the right to refuse to engage in the trial if he/she did not want to. In order to protect the privacy of patients, we used anonymized numbers to code these patients.

## 3 RESULTS

### 3.1 Patients characteristics

**FIGURE 1:**
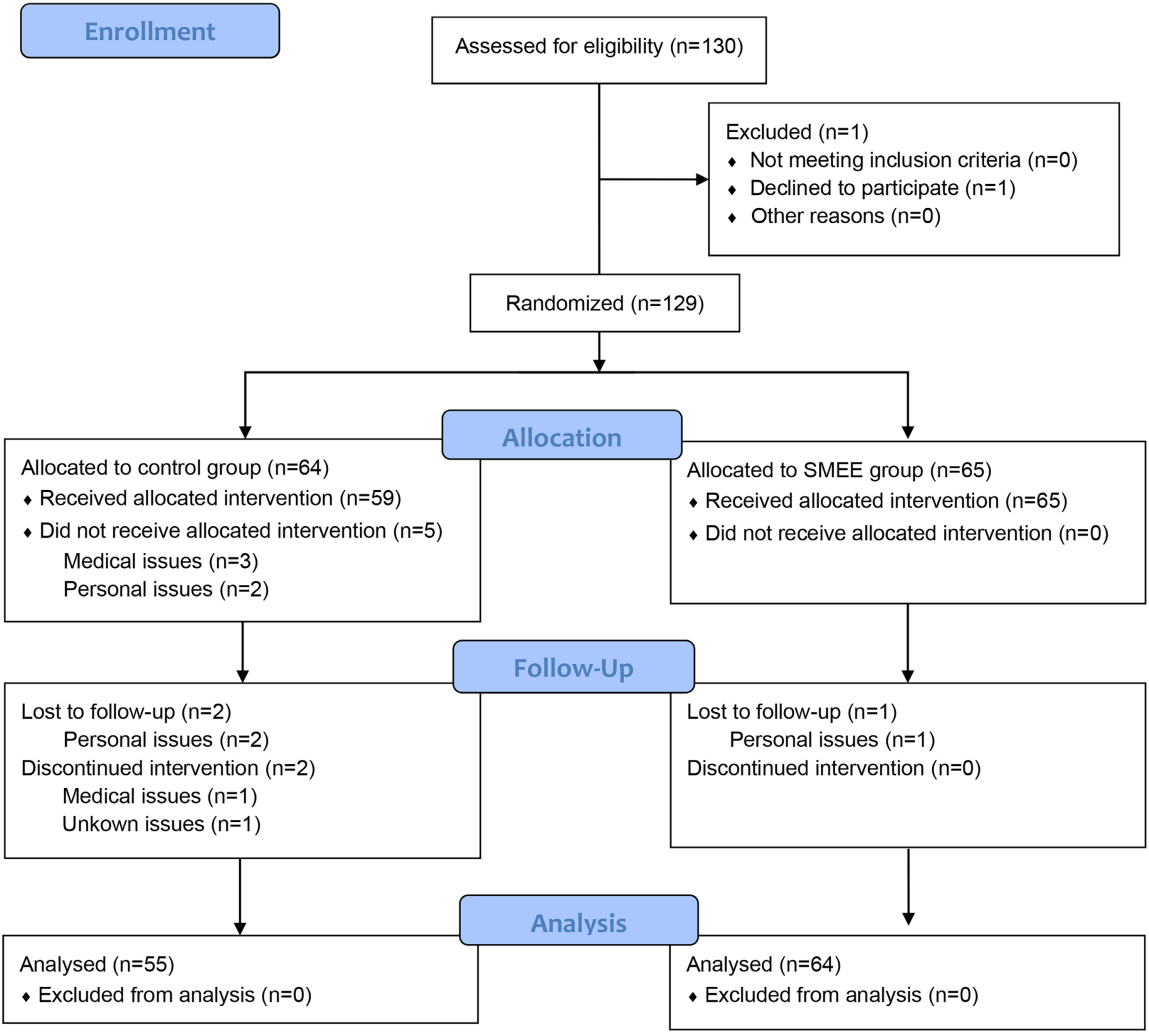
CONSORT flow diagram.

A total of 130 eligible patients with GC in the Department of Oncology of Ruijin Hospital (1 patient declined to participate after screening) were initially enrolled and randomly assigned into the SMEE group (*n* = 65) and the control group (*n* = 64). 5 patients did not receive allocated intervention due to medical (*n* = 3) or personal issues (*n* = 2). 3 patients were lost to follow‐up because of personal issues, and 2 patients had discontinued intervention. 119 patients (64 in the SMEE group and 55 in the control group) completed the assigned intervention (Figure 1). The percentages of protocol deviations were 1.5% and 14.1% in the SMEE and control groups, respectively. Male proportions were 53.1% and 53.8%, and mean ages were 60 and 58 years in the SMEE and control groups, respectively. Baseline characteristics including sex, age, education level, cancer TNM stage, treatment, and catheters use did not differ significantly between the two groups in both the intention‐to‐treat and per‐protocol analyses (Table 1).

**TABLE 1:** Participants' characteristics.

	Intention‐to‐treat			Per protocol		
	SMEE group	Control group	*p*	SMEE group	Control group	*p*
*n*	65	64		64	55	
Gender						
Male	35 (53.8)	34 (53.1)	0.935	34 (53.1)	30 (54.5)	0.877
Age (years)	58.4 ± 10.0	57.2 ± 10.6	0.499	58.2 ± 10.0	55.6 ± 10.2	0.150
Education						
Primary school or lower	7 (10.8)	7 (10.9)	0.988	7 (10.9)	7 (12.7)	0.964
Junior high school	18 (27.7)	20 (31.3)		18 (28.1)	15 (27.3)	
High school/technical secondary school	15 (23.1)	15 (23.4)		14 (21.9)	14 (25.5)	
College/university	21 (32.3)	18 (28.1)		21 (32.8)	15 (27.3)	
Above college degree	4 (6.2)	4 (6.3)		4 (6.3)	4 (7.3)	
Live alone						
Yes	12 (18.5)	8 (12.5)	0.350	8 (12.5)	6 (10.9)	0.483
Stage						
II	10 (15.4)	11 (17.2)	0.956	10 (15.6)	8 (14.5)	0.593
III	14 (21.5)	13 (20.3)		13 (20.3)	10 (18.2)	
IV	41 (63.1)	40 (62.5)		41 (64.1)	37 (67.2)	
Treatment method						
Chemotherapy	10 (15.4)	8(12.5)	0.636	10 (15.6)	8 (15.1)	0.870
Gastrectomy and chemotherapy	55 (84.6)	56 (87.5)		54 (84.4)	47 (84.9)	
No. of chemotherapy drugs						
1	22 (33.8)	18 (28.1)	0.782	22 (34.4)	18 (32.7)	0.813
≥2	43 (66.2)	46 (71.9)		42 (65.6)	37 (67.3)	
Catheter use						
Yes	31 (47.7)	24 (37.5)	0.242	31 (48.4)	21 (38.2)	0.261

Abbreviation: SMEE, self‐designed metabolic equivalent exercises.

### 3.2 Fatigue status

The total RPFS fatigue status and subscale scores before the metabolic equivalent exercises intervention were not significantly different between the two groups (all *p* > 0.05). After 3 months of intervention, the total fatigue score for the patients in the SMEE group (2.86 ± 1.75) was significantly lower than that for those in the control group (4.65 ± 1.29), and there were significant improvements in affective meaning (0.83 ± 0.92 vs. 1.13 ± 0.77, *p* = 0.044) and sensory (0.70 ± 0.71 vs. 1.00 ± 0.54, *p* < 0.001) subscales (Table 2).

**TABLE 2:** Fatigue status as assessed using the Revised Piper Fatigue Scale (RPFS).

Time	Group	Behavioral/severity subscale	Affective meaning subscale	Sensory subscale	Cognitive mood subscale	Total score
Baseline	SMEE group	1.36 ± 0.74	1.19 ± 0.75	1.11 ± 0.67	0.72 ± 0.623	4.39 ± 1.68
	Control group	1.27 ± 0.68	1.18 ± 0.86	0.95 ± 0.63	0.93 ± 0.77	4.33 ± 2.00
	*t*	0.66	0.038	1.349	−1.63	0.188
	*p*	0.511	0.969	0.180	0.106	0.851
3 months	SMEE group	0.8 ± 0.67	0.83 ± 0.92	0.7 ± 0.71	0.39 ± 0.68	2.86 ± 1.75
	Control group	1.45 ± 0.81	1.13 ± 0.77	1.00 ± 0.54	0.65 ± 0.62	4.65 ± 1.29
	*t*	−4.836	−1.906	−2.587	−2.203	−6.287
	*p*	0.057	0.044*	<0.001*	0.874	0.009*

Abbreviation: SMEE, self‐designed metabolic equivalent exercises.

* *p* < 0.05.

### 3.3 Quality of life

At baseline, the 5 functional and 9 symptoms dimensions assessed using the Chinese version of the EORTC QLQ‐C30 were comparable between the 2 groups (all *p* > 0.05). After 3 months of intervention, among the 5 functional dimensions, somatic (2.00 ± 0.27 vs. 1.31 ± 0.26, *p* < 0.001), emotional (2.67 ± 0.58 vs. 2.07 ± 0.48, *p* < 0.001), and social (3.23 ± 0.58 vs. 1.64 ± 0.51, *p* < 0.001) functioning were significantly improved in the SMEE group compared to the control group. However, role or cognitive function was not significantly different between the 2 groups (Table 3).

**TABLE 3:** Quality of life (functional dimensions) as assessed using the EORTC QLQ‐C30 scales.

Time	Group	Somatic function	Character function	Emotional function	Cognitive function	Social function
Baseline	SMEE group	2.19 ± 0.42	1.53 ± 0.58	1.49 ± 0.52	2.05 ± 0.48	2.32 ± 0.47
	Control group	2.30 ± 0.61	1.35 ± 0.47	1.49 ± 0.55	2.15 ± 0.49	2.45 ± 0.65
	*t*	3.276	−1.830	0.013	1.145	−1.916
	*p*	0.128	0.070	0.990	0.254	0.580
3 months	SMEE group	2.00 ± 0.27	1.53 ± 0.58	2.67 ± 0.58	2.23 ± 0.44	3.23 ± 0.58
	Control group	1.31 ± 0.26	1.35 ± 0.47	2.07 ± 0.48	1.96 ± 0.56	1.64 ± 0.51
	*t*	−14.202	−1.83	−6.059	−2.784	−15.95
	*p*	<0.001*	0.070	<0.001*	0.060	<0.001*

Abbreviation: SMEE, self‐designed metabolic equivalent exercises.

* *p* < 0.05.

Regarding the symptoms dimension scores, fatigue (consistent with the findings based on RPFS scores), nausea and vomiting, shortness of breath, constipation, and diarrhea symptoms were significantly improved (all *p* < 0.05) after 3 months of metabolic equivalent exercises, which are aerobic exercises and which improve metabolism. However, the scores for the dimensions of pain, insomnia, loss of appetite, and financial difficulties were not significantly different between the 2 groups (Table 4).

**TABLE 4:** Quality of life (symptoms dimensions) as assessed using the EORTC QLQ‐C30 scales.

Time	Group	Fatigue	Nausea, vomiting	Pain	Shortness of breath	Insomnia	Loss of appetite	Constipation	Diarrhea	Economic hardship
Baseline	SMEE group	2.61 ± 0.63	2.24 ± 0.60	2.12 ± 0.44	1.67 ± 0.56	1.89 ± 0.62	2.12 ± 0.86	1.47 ± 0.53	1.53 ± 0.73	1.56 ± 0.59
	Control group	2.51 ± 0.51	2.09 ± 0.42	2.10 ± 0.44	1.73 ± 0.49	1.9 ± 0.58	2 ± 0.64	1.63 ± 0.62	1.47 ± 0.69	1.75 ± 0.73
	*t*	0.95	1.60	0.21	−0.57	−1.66	0.91	−1.57	0.45	−1.52
	*p*	0.342	0.112	0.835	0.573	0.871	0.372	0.124	0.686	0.132
3 months	SMEE group	2.13 ± 0.64	2.26 ± 0.64	2.00 ± 0.25	1.22 ± 0.42	1.64 ± 0.73	2.36 ± 1.04	1.80 ± 0.65	1.39 ± 0.61	1.66 ± 0.63
	Control group	2.93 ± 0.44	2.55 ± 0.60	2.16 ± 0.50	1.62 ± 0.62	1.62 ± 0.73	2.33 ± 0.88	2.71 ± 0.71	1.82 ± 0.70	1.73 ± 0.62
	*t*	−8.039	−2.51	−2.20	−4.16	0.18	0.18	−7.27	−3.58	−0.62
	*p*	<0.001*	0.014*	0.310	<0.001*	0.875	0.861	<0.001*	0.001*	0.536

Abbreviation: SMEE, self‐designed metabolic equivalent exercises.

* *p* < 0.05.

### 3.4 Success rate and completion rate

The metabolic equivalent exercise intervention had high success and completion rates, all of which were ≥50% (Table 5). In the SMEE group, 68.2% of patients had a success rate of 81%–100%, and 50.0% had a completion rate of 81%–100%.

**TABLE 5:** Success rates and completion rates of metabolic equivalent exercise.

Group	Success rate (*n*, %)			Completion rate (*n*, %)		
	50%–70%	61%–80%	81%–100%	50%–70%	61%–80%	81%–100%
SMEE group (*n* = 64)	22 (34.3)	27 (42.2)	15 (23.4)	9 (14.1)	23 (35.9)	32 (50.0)
Control group (*n* = 55)	13 (23.6)	26 (47.3)	16 (29.1)	12 (21.8)	17 (30.9)	26 (47.3)

Abbreviation: SMEE, self‐designed metabolic equivalent exercises.

## 4 DISCUSSION

Cancer‐related fatigue (CRF) is one of the important factors affecting the quality of life of patients with gastric cancer (GC). 75% to 96% of patients undergoing chemotherapy have different degrees of CRF. Exercise is an effective nonpharmacological intervention to treat CRF and can effectively alleviate CRF symptoms. However, there are few reports on the effective use of exercise intervention in GC patients, and there have been few randomized interventional studies on CRF in patients with GC in China or other countries. This is the first randomized controlled trial (RCT) to investigate the effect of the Self‐designed Metabolic Equivalent Exercises (SMEE), whose design was based on the exercise oncology prescription framework proposed by Sasso et al.^22^ and in line with the principles of individualization, targeting, rest and recovery, and progressive overload, on CRF in Chinese patients with GC.

In our RCT, the self‐programmed metabolic equivalent manipulation as an exercise intervention effectively reduced the degree of cancer‐caused fatigue and improved quality of life in patients with GC. After 3 months, the total RPFS score of the SMEE group was significantly lower than that of the control group (2.86 ± 1.75 vs. 4.65 ± 1.29), with significant improvements in affective meaning (0.83 ± 0.92 vs. 1.13 ± 0.77) and sensory (0.70 ± 0.71 vs. 1.00 ± 0.54) subscales; in the SMEE group, QLQ‐C30 scores in somatic (2.00 ± 0.27 vs. 1.31 ± 0.26), emotional (2.67 ± 0.58 vs. 2.07 ± 0.48), and social (3.23 ± 0.58 vs. 1.64 ± 0.51) function were significantly higher than those in the control group, with significant improvements in fatigue, nausea/vomiting, shortness of breath, constipation, and diarrhea dimensions.

Notably, these findings should be carefully interpreted considering the sample characteristics. In the SMEE and control groups, male proportion was 53%–55%, with a mean age of 56–58 years. Most of the patients (58%–62%) received an education of high school or above. Patients with Stage IV disease composed the majority (63%–67%). All patients underwent chemotherapy, and most of the patients (66%–72%) used at least 2 chemotherapeutic drugs. The majority of the patients (84%–88%) also underwent gastrectomy.

In this study, the fatigue status of the SMEE group was significantly better than that of the control group after the 3‐month metabolic equivalent exercise intervention, and the total fatigue score decreased in relation to that for the control group, similar to the score for the symptoms dimension of the quality of life scale. In addition to improving CRF, exercise can also effectively improve cardiopulmonary function and mental state, allowing patients to maintain an optimistic and positive attitude. A cohort study of more than 30,000 individuals showed that any intensity of exercise during leisure time can prevent the occurrence of depression.^23^ In this study, the emotional state dimension score in the fatigue scale and the emotional function dimension score in the quality of life scale verified that conclusion. In addition, a meta‐analysis showed that aerobic exercise and resistance exercise can improve the symptoms of cancer patients, including fatigue, nausea and vomiting, pain, dyspnea, insomnia, loss of appetite, constipation, and diarrhea.^24^

When considering the feasibility of cancer patients exercising, a study suggested that physical exercise is feasible and accessible.^25^ A study^26^ of 572 patients with terminally ill cancer showed that 528 patients (92%) were able to perform at least one unit of exercise (average 4.2 units per patient) and that physical exercise was the most common and feasible modality for 50% of patients. Therefore, when the physical strength of a cancer patient is adequate, the patient should be encouraged to actively exercise. Exercise is not only an activity but also an effective treatment method.

Although exercise helps to improve the health outcomes of cancer patients, there are still many obstacles that affect participation in exercise.^19^ From the perspective of the completion rate and compliance rate of exercise in this study, the completion rate represents the compliance of the patients with the exercise program. Only 50% of the SMEE group was able to complete more than 80% of the planned exercise time; for the control group, the rate was even lower (47.3%). The compliance rate indicates whether a patient's exercise intensity generated the target heart rate. There was a significant difference between the SMEE group and the control group, indicating that the intensity of metabolic equivalent exercise met the needs of moderate to high‐intensity exercise in the patients.

In clinical practice, it has been found that cancer patients cannot accurately assess the type and intensity of exercise, lack accurate exercise guidance during hospitalization, lack exercise habits, lack exercise skills, and fear the negative effects of exercise on treatment and the disease. These obstacles prevent them from taking the right and effective exercise to relieve CRF.^21^ Therefore, clinicians must first help patients establish the awareness that exercise, when implemented correctly, can improve CRF. In addition, patients should be given correct guidance including exercise types, intensity, frequency, duration, precautions, et al.^27^ Metabolic equivalent exercise programs are developed based on the characteristics of cancer patients by considering factors such as intubation and fatigue. Such programs solve the problem of patients not knowing what kind of exercise to engage in and ensure the correct level of exercise, thus encouraging patients to exercise more.

After patients are discharged from the hospital, they can continue exercising with guidance from videos. Metabolic equivalent exercises are easy to perform, with high patient acceptance, wide dissemination and significant results. In addition to exercise, through the adoption of effective guidelines, the current knowledge on the best management of CRF can be transformed into clinical practice, and clinical application can be further promoted through guideline adjustments, professional education and integration with existing practices.^28^

It has been suggested that exercise targets and improves almost all imaginable outcomes of cancer patients,^16^ and the results herein have confirmed that proposition. The results for the 4 dimensions, that is, physical function, emotional function, social function, and symptoms, of the patients all showed that exercise significantly improved the health outcomes and increased quality of life. Sasso et al.^17^ noted that although there is considerable heterogeneity in more than 100 published exercise intervention studies involving cancer patients, it is generally believed that exercise is related to objective physiological indicators (such as cardiopulmonary function, physical function, and body composition) as well as positive changes in patient‐reported results (e.g., fatigue, sleep quality, and physical strength). Epidemiological studies have shown that physical activity during leisure time can reduce the risk of at least 13 different types of cancers.^20^ It is necessary to strengthen personal health responsibilities, improve the health literacy of all people, guide the formation of a self‐disciplined healthy lifestyle, effectively control the factors that affect healthy life behaviors, and form a social atmosphere that celebrates health, pursues health, and promotes health. Therefore, exercise has far‐reaching significance for the primary prevention of tumors.

### 4.1 Limitations

Several limitations of this study warrant consideration. Firstly, since many of the patients underwent gastrectomy in other hospitals, detailed information on gastrectomy was not available. There are 24 items according to the CTCAE 5.0, and the collection of relevant information was not done during this study implementation; this will be carefully addressed in future studies. Secondly, participants with specific characteristics were recruited from a tertiary hospital in Shanghai, China, and the findings might not be adequately generalizable to the other population. A multicentric RCT is warranted. Thirdly, when the patients performed the metabolic equivalent exercises, they themselves recorded the exercise date, exercise duration, and heart rate. There was a lack of objective monitoring by caregivers. For studies in the future, electronic wearable devices could be used to more accurately record and to more effectively monitor exercise information in a timely manner. Fourthly, while not the endpoints of this RCT, the long‐term effects and oncologic and survival impacts of the exercises were not determined.

## 5 CONCLUSIONS

The self‐designed metabolic equivalent exercise program, which is a new form of exercise performed at intensities and which was quantitatively tailored for each individual with no restrictions on field or time, was applicable with good compliance and could effectively improve the fatigue status and quality of life in patients with GC through exercise. Adequate exercise program, which could effectively improve health‐related outcomes, should be implemented as an integral and standardized component of palliative care. Future efforts should focus on developing more personalized exercise programs and applying wearable devices for patients with GC.

## AUTHOR CONTRIBUTIONS

**Xiao Xin:** Conceptualization (equal); data curation (equal); formal analysis (equal); funding acquisition (equal); investigation (equal); methodology (equal); project administration (equal); resources (equal); software (equal); supervision (equal); validation (equal); visualization (equal); writing – original draft (equal); writing – review and editing (equal). **Lei Huang:** Conceptualization (equal); data curation (equal); formal analysis (equal); funding acquisition (equal); investigation (equal); methodology (equal); project administration (equal); resources (equal); software (equal); supervision (equal); validation (equal); visualization (equal); writing – original draft (equal); writing – review and editing (equal). **Qi Pan:** Data curation (equal); formal analysis (equal); investigation (equal); methodology (equal); validation (equal); visualization (equal); writing – review and editing (equal). **Jun Zhang:** Conceptualization (equal); funding acquisition (equal); investigation (equal); project administration (equal); resources (equal); supervision (equal); validation (equal); writing – review and editing (equal). **Weiguo Hu:** Conceptualization (equal); funding acquisition (equal); investigation (equal); project administration (equal); resources (equal); supervision (equal); validation (equal); writing – review and editing (equal).

## FUNDING INFORMATION

This study was supported by Ruijin Hospital Nursing Research Program (RJHK‐2020‐16), Nursing Plateau Construction Fund (hlgy1904kygg), and special project of Medical Center on Aging of Ruijin Hospital, MCARJH (GB202103). The funders had not been involved in study design; in the collection, analysis, or interpretation of data; in the writing of the report; or in the decision to submit the paper for publication.

## CONFLICT OF INTEREST STATEMENT

The authors declare that they have no conflicts of interest.

## ETHICS STATEMENT

This study was approved by the Ethics Committee of Ruijin Hospital.

## CLINICAL TRIAL INFORMATION

This randomized controlled trial was registered in clinicaltrials.gov (NCT05401045).

## Supporting information


**Video S1:** Self‐designed Metabolic Equivalent Exercises (SMEE).

## Data Availability

Data are available upon reasonable request from the corresponding authors with permission of the institution.
